# The Cytotoxic, Apoptotic Induction, and Cell Cycle Arrest Activities of *Solanum nigrum *L. Ethanolic Extract on MCF-7 Human Breast Cancer Cell

**DOI:** 10.31557/APJCP.2020.21.12.3735

**Published:** 2020-12

**Authors:** Churiyah Churiyah, Sri Ningsih, Firdayani Firdayani

**Affiliations:** *Center for Pharmaceutical and Medical Technology, Agency for the Assessment and Application of Technology, LAPTIAB Building 611, Puspiptek Area, Serpong, Tangerang-Selatan, Indonesia. *

**Keywords:** Solanum nigrum L- MCF-7, cytotoxicity, apoptotic, isoflavone

## Abstract

**Objective:**

The purpose of this research was to evaluate the cytotoxic, cell cycle arrest, and apoptotic induction activities of the fruit of *S. nigrum* L. ethanolic-70% extract against MCF-7 human breast cancer cell.

**Methods:**

*S. nigrum *L. ripe fruit was blended and macerated with ethanol 70% and the filtrate was evaporated. The semisolid extract was then analyzed phytochemically. Cytotoxic analysis was performed using MCF-7 cancer and Vero normal cell by MTT method and followed by apoptotic and cell cycle arrest analysis using flow cytometry.

**Results:**

The phytochemical analysis resulted that extract contained total phenolic and flavonoid compounds with the level of 1.545±0.080% and 0.212±0.002%, respectively. Glycitin was the highest level of isoflavone compound, namely, 375.0844 mg/100 g extract. The cytotoxic evaluation revealed that the extract exhibited a selectively toxic effect between cancer and normal cell. The extract inhibited MCF-7 proliferation with IC50 value about 40.77±4.86 μg/mL and conversely toward Vero cell at lower cytotoxic activity with an IC50 value of 298.96±27.28 μg/mL. Evaluation of MCF-7 cell cycles demonstrated that the extract arrested the cell cycle in the S phase and continued to the G2/M phase at the half of the IC50 value. The extract induced apoptotic of MCF-7 cell about 43.31% in which this activity was nearly the same with doxorubicin as a positive control (59.14%). However, solamargine was predicted as the most active anticancer compounds by a molecular docking study so that it was suggested to measure the level of this compound.

**Conclusion:**

It can be concluded that the fruit of *S. nigrum* L. ethanolic-70% extract demonstrated cytotoxic activity toward MCF-7 breast cancer cell and nontoxic on Vero normal cell. Solamargine was predicted as the most active anticancer compound. This extract had an opportunity to be developed as a potential anticancer agent to overcome breast cancer diseases.

## Introduction

Breast cancer is a type of cancer with the highest prevalence in women and causes high mortality rates (Iqbal et al., 2017). Conventional treatment of cancer with chemotherapy often has elicited some side effects. An effort to find new potential anticancer compounds with specificity on tumor cells without harmful toward normal cells is challenging (Jamalzadeh et al., 2017). Various natural resources, including medicinal plants, have become targets for exploring new anticancer compounds with the lowest side effects. Many active compounds have been proven capable of apoptosis induction in several cancer cells (Ali et al., 2012). Therefore, it is necessary to develop more effective and less toxic anticancer drugs immediately.

*Solanum nigrum* L. is one of the Solanaceae family. Some locales have been using the ripe fruit as vegetables, while the stems and leaves are useful as traditional medicine. This plant obtained various chemical compounds, including glycolalkaloids, polyphenolic, polysaccharide, and glycoprotein, which showed some activities such as cytotoxic, scavenging radical compound, anti-inflammatory and anticancer (Chauhan et al., 2012). The methanol extract of fruits has been evaluated by cytotoxic activity on the HeLa service cancer cell (Patel et al., 2009). It was claimed that the polyphenol-enriched extract of *S. nigrum* L. ripe fruit inhibited the cell cycle process and induced apoptosis on Normal and cancer cells of the human prostate (Nawab et al., 2012). A class of glycoalkaloids, known as solasodine rhamnosyl glycosides, were secondary metabolites found in Solanaceae that observed for inducing apoptosis in cancer cells without developing resistance of those cancer cells also. These compounds exerted a positive immune response towards cancer cells (Cham, 2013). The methanolic extracts of leaves and stems exhibited anticancer activity on various cancer cells such as PC3 prostate, HeLa, mouse embryo fibroblast T3T NIH, and Wistar hepatocyte CC-1 (Moglad et al., 2014).

MCF-7, a luminal cancer cell type, becomes an appropriate cell line model in the effort for discovering new breast cancer entities and also provides many data for practical knowledge compared to other types of cells. It is claimed that several genes are involved in controlling this cell growth, such as estrogen and progesterone receptor (ER and PR) and also by plasma membrane-associated growth factor receptors, namely, HER2 and EGFR (human epidermal growth factor receptor-2 and epidermal growth factor receptor) (Comsa et al., 2015). EGFR is a kind of receptor comprised of a tyrosine kinase that takes the central part in all living cells, especially for differentiation, normal development, and proliferation. Some previous studies proved that EGFR involved in carcinogenesis steps of several tumor types, and it had been discovered that EGFR inhibitor compounds that had overcome carcinoma disorder (Xu et al., 2010).

The purpose of this research was to evaluate the cytotoxic activity of the fruit *S. nigrum* L.-derived ethanolic extract on breast cancer cell MCF-7 by MTT system and to analyze the inducing apoptosis and inhibiting cell cycle of these cells by flow cytometry. The phytochemical evaluation was conducted to measure the level of a secondary metabolite of the extract. Besides, to predict this anticancer mechanism, it was also performed a simulation in silico with molecular docking technique toward EGFR.

## Materials and Methods

*Preparation of S. nigrum L. ethanolic extract*

The *S. nigrum* L. ripe fruits obtained from the local market were washed with running water to remove the sticking dirt particles. After being air-dried, they were grounded and macerated with 70% ethanol solvent. The filtered macerate was evaporated in a vacuum condition at 40°C until the viscous extract obtained.

*Phytochemical screening of S. nigrum L. ethanolic extract*

The qualitative screening of phytochemicals of *S. nigrum* L. fruit extract was performed to identify the chemical constituents (alkaloid, terpenoid/steroid, flavonoid, saponin, tannin, quinone, essential oil, and coumarin) based on the previous method (Ashrafudoulla et al., 2016).

*Total polyphenol and total flavonoid determination*

Determination of total polyphenol using the Folin Ciocalteu reagent and total flavonoid using AlCl3 and CH3COOK solution followed the previous method (Abd Ghafar et al., 2010).

*Isoflavone compound determination using HPLC*

The isoflavone compounds measured in S. Nigrum L. extract were daidzin, glycitin, genistin, daidzein, glycitein, and genistein using Hitachi HPLC machine based on previous paper (Collison, 2008) with slight modification. The HPLC analysis was carried out using LiChocart 60 RP-Select B, 250-4 µm column, 10 uL volume of sample injection, and at the flow rate of 0.8 mL/min. The elution was used gradient system that mobile phases A and B were comprised of aquadest-10%phosphat acid and acetonitrile, respectively. The standard stock solution was prepared by mixing daidzin 10 mg, glycitin 5 mg, genistin 10 mg, daidzein 10 mg, glycitein 5 mg, and genistein 20 mg tared 5 mL volumetric flask, dissolved to volume with DMSO. Working solutions were prepared by diluting the stock solution into a range concentration (0.044, 0.094, 0.143, 0.189 and 0.237 mg/mL) using 40% acetonitrile solution and then added to each concentration with 0.025 mL of internal standard apigenin 2 mg/ml in DMSO. The sample solution was dissolving 51.6 mg in 1 mL DMSO and then added with internal standard. The peak of each compound was detected with a UV detector at 260 nm. The concentration of each marker compound was calculated from the standard curve equation (y=ax+b) derived from the curve standard plotting between each concentration against the ratio of peak area each compound to an internal standard. The concentration of each marker compound was reported as mg/100 g sample weight.

*Cytotoxicity assay*

The cytotoxicity test was conducted on MCF-7 cancer cell and Vero normal cell that both of these cells were courtesy of Prof. Dr. Edy Meyanto, Faculty of Pharmacy, Gadjah Mada University, Indonesia. The MCF-7 cell was cultured in RPMI 1640 medium augmented with 10% heat-inactivated Fetal Bovine Serum (FBS), 1% antibiotic of penicillin-streptomycin, and incubated in a 5% CO_2_ incubator at 37°C until confluent. The confluent cell was harvested by trypsin. About 100 ul of cell suspension with the amount of 5x10^3^ cell was plated into each well of the 96 well microplates. After being incubated for 24 h, the medium was replaced with 100 μL of serial concentrations of samples test, and the plate was then incubated for 24 h anymore. The sample solutions were discarded, the plate was washed twice with 100 uL of PBS, and then an amount of 100 μL medium containing 0.05 mg/L of MTT was added to each well. After being incubated for four h, 100 μL 10% SDS solution was added and followed incubating overnight in the dark at room temperature. The color intensity was recorded using a microplate reader at 570 nm. The percentage of cell growth inhibition was calculated, and the concentration of the sample that inhibited 50% of cell growth (IC_50_) was derived from the dose-response curves of the cell.

*Flow cytometry cell cycle arrest and apoptotic cell analysis*

The MCF-7 cells were plated on 24 well plates with an initial total cell of 3x10^4^ cells/well, and then incubated in 5% CO_2_ incubator for 24 hours, and following 24 hours treatment with *S. nigrum* L. extract at half of IC_50_ concentration. Furthermore, the cells were separated by centrifugation at 1,500 rpm for 5 minutes, and then the pelleted cell was fixed with 500 µL of 70% ethanol in 4^o^C for 15 minutes. After removing ethanol by centrifugation at 1500 rpm for 5 min, cell pellet obtained was added with 450 µLPBS and 50 µLpropidium iodide (Sigma P3566) (1 mg PI/mL of deionized water), incubated at room temperature in dark conditions for 30 minutes. Then cells were evaluated using flow cytometry (BD AccuriTM C6 Plus). Doxorubicin as a positive control was used at half of the IC_50_ concentration, and as a negative control was prepared by cells treated with medium only. 

*Molecular Docking Simulation*

Molecular docking simulation was conducted to predict the inhibitory mechanism action of *S. nigrum* L. toward EGFR using Molegro Virtual Docker ver 6.0. The 3D crystal structure of EGFR was used as a receptor in docking study, and active compounds of *S. nigrum* L. were selected to be docked into the receptor. The validation of the docking protocol settings was done by re-docking the extracted co-crystallized ligand reference from the 3D structure using the same protocol for the docked compounds. The docking protocol was performed at the first step through re-docking of the extracted co-crystallized ligand reference. The protocol setting was valid if the RMSD value less than 2Å (Hevener, 2009).

*Data analysis*

All of the experiments were carried out in triplicate, and the results were expressed as mean±s.d. The Student’s T-test with Microsoft Excel program was used for statistical analysis. The difference was considered significant if the P-value was less than 0.05 (P<0.05).

## Results

*Phytochemical analysis of ethanolic extract of S. nigrum L.*

The phytochemical screening of *S.* *nigrum* L. extract contained several group compounds, namely were alkaloid, flavonoid, saponin, tannin, steroid/triterpenoid, and coumarin but did not found any quinone and volatile oil. The measurement of total phenolic and total flavonoid compounds demonstrated that each group compound’s level was 1.545±0.080% and 0.212±0.002%, respectively. 

**Figure 1:**
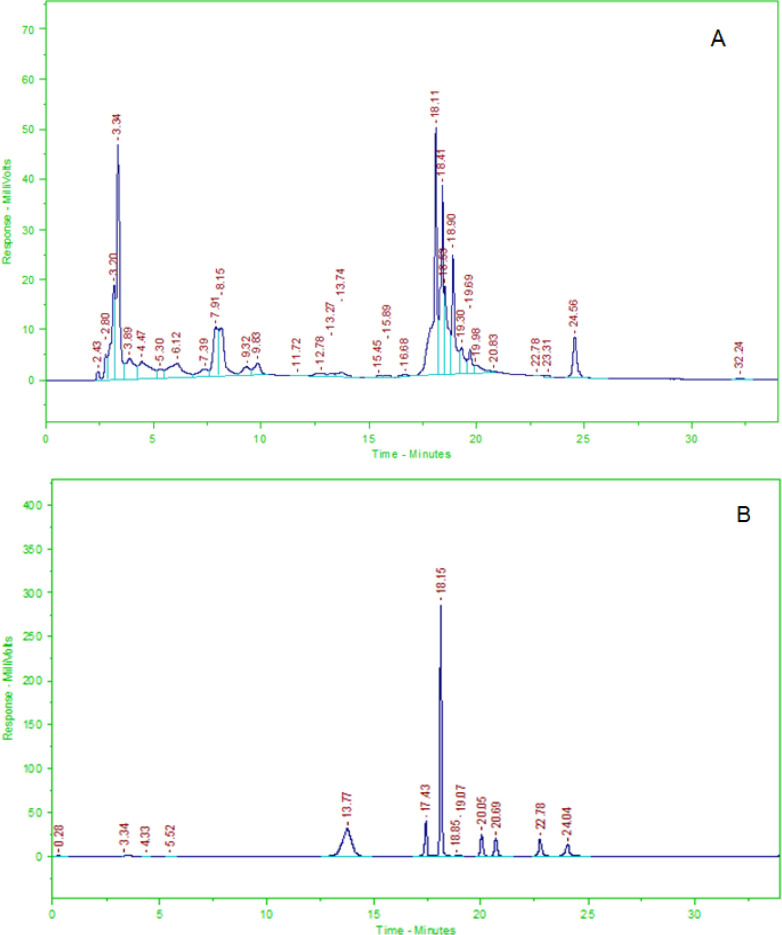
HPLC Chromatograms of the Fruits of *S. nigrum* L. ethanolic Extract (A) and the standard of isoflavone compounds (B). The order of each peak from left to right was daidzin - glycitin - genistin - daidzein - glycitien - genistein – apigenin (B)

HPLC chromatogram of *S. nigrum* L. extract was depicted in Figure 1. It showed that the peaks of each isoflavone compound in the order from left to right were daidzin - glycitin - genistin - daidzein - glycitien - genistein - apigenin with RT 13.77, 17.43, 18.15, 20.05, 20.69, 22.78, and 24.04, respectively. The use of standard internal apigenin in this measurement technique was intended as a correction if there was a shift of each compound peak due to technical factors. The calculation of each isoflavone compound in the *S. nigrum* L. extract demonstrated that glycitin was the highest level, followed by genistin, daidzein, and genistein 375.0844, 109.2039, 15.6771, and 1.0029 mg/100 g semisolid sample weight, respectively. However, out of two compounds were not detected in the solanum extract, namely, daidzein and glycitein. 

*Cytotoxicity assay*

**Figure 2:**
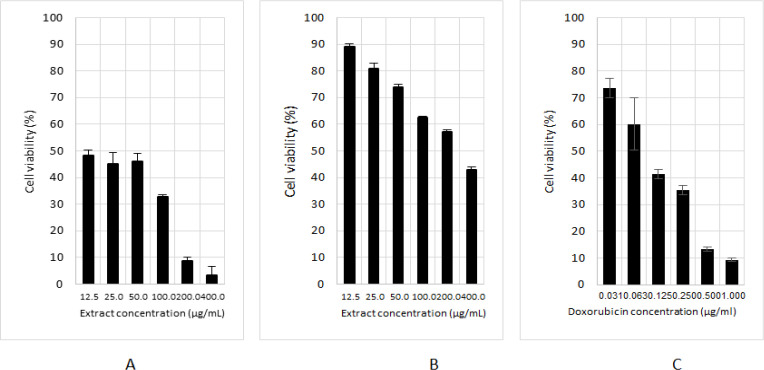
The Cytotoxic Activities of the Fruit of *S. nigrum* L. ethanolic extract. (A) *S. nigrum* L. extract on the MCF-7 cancer cell, (B) *S. nigrum* L. extract on Vero normal cell, (C) doxorubicin on MCF-7 cell

The result of MTT assay of *S. nigrum* L. extracts toward MCF-7 and Vero normal cell as depicted in Table 1 and Figure 2. The principle assay of MTT is that viable cells can cleavage tetrazolium compound (MTT) into an insoluble blue-colored formazan by succinate dehydrogenase mitochondrial enzyme of viable cells. Otherwise, the dead cell cannot reduce tetrazolium. Therefore, the stronger the tested sample suppressed cell growth, the lower the intensity of blue color will be produced (Mahadev et.al., 2015). The cytotoxic activity of *S. nigrum* L. extracts against both cancer and normal cancer cell demonstrated dose-dependent property, where the increase of extract concentration followed with the decrease of cell viabilities (Figure 2). Interestingly, this extract was capable of selectively inhibiting cellular proliferation of MCF-7 cancer cell with IC50 value about 40.77±4.86 μg/mL, and conversely, this extract less exhibited a cytotoxic effect on Vero normal cell with IC50 value of 298.96±27.28 μg/mL. Index selectivity of this extract was 7.33 in which it was mean that the extract was more toxic toward MCF-7 cancer cell than normal cell Vero. This result indicated that the extract has selectively properties that killed the cancer cell but was not toxic to the normal cell. This property is desirable in the exploration of anticancer agents.

*Cell cycle arrest and apoptotic analysis*

**Figure 3:**
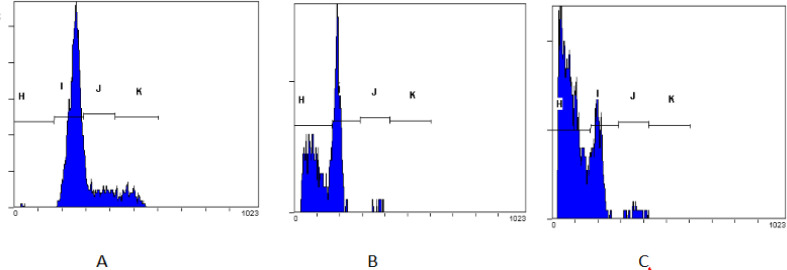
The Cytogram of MCF-7 Cancer Cell. (A) Cells treated with medium control. (B) Cells treated with *S. nigrum* L. extract. (C) Cells treated with doxorubicin

**Figure 4:**
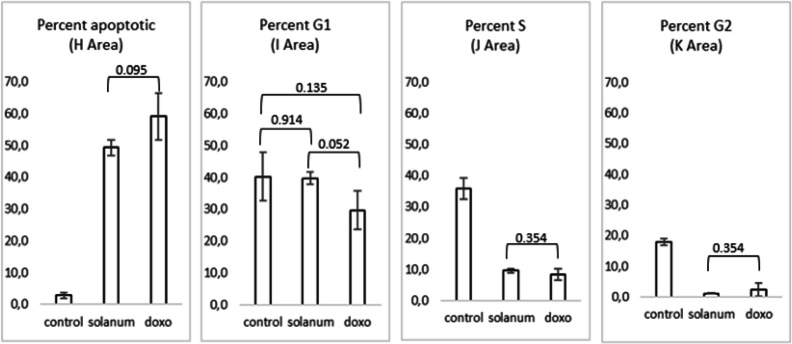
Distribution of MCF-7 Cell at Each stage of Cell Cycles. The value was an average from triplicate. Experiments conducted at the final concentration of *S. nigrum* L. extract and doxorubicin were half of IC_50_, 20, and 0.07 μg/mL, respectively. The control cell was treated with the medium. P-value > 0.05 = no different significantly

The assay’s principle is that the propidium Iodide (PI) will bind to DNA by intercalation between the base with little or no base sequence preference. This PI is a fluorescent compound which very soluble in water, and this dye cannot pass through intact living cell membranes. Therefore, the presence of high fluorescence intensity indicates membrane integrity. The activities of *S nigrum *L. fruit extract in inducing apoptosis and inhibiting cell cycle using flow cytometry were presented in Figures 3 and 4. The result of flow cytometry analysis revealed that the strength of *S. nigrum* L. extract induced MCF-7 cell apoptotic was not different significantly compared to doxorubicin (p>0.05) (Figure 4). This extract arrested the cell cycle in the S phase and continued to the G2/M phase. The cell cycle study showed that both extract and doxorubicin-treated MCF-7 demonstrated that the number of cells expressed by area for both S and G2/M phase was not different significantly. *S. nigrum* L. extract at the final concentration of 20 μg/mL delivered the ability to suppress the cell cycle with similar strength as doxorubicin (0.07 μg/mL). This result revealed that *S nigrum *L. extract had potential in inhibiting the MCF-7 cell cycle.

*Molecular Docking Simulation*

**Figure 5:**
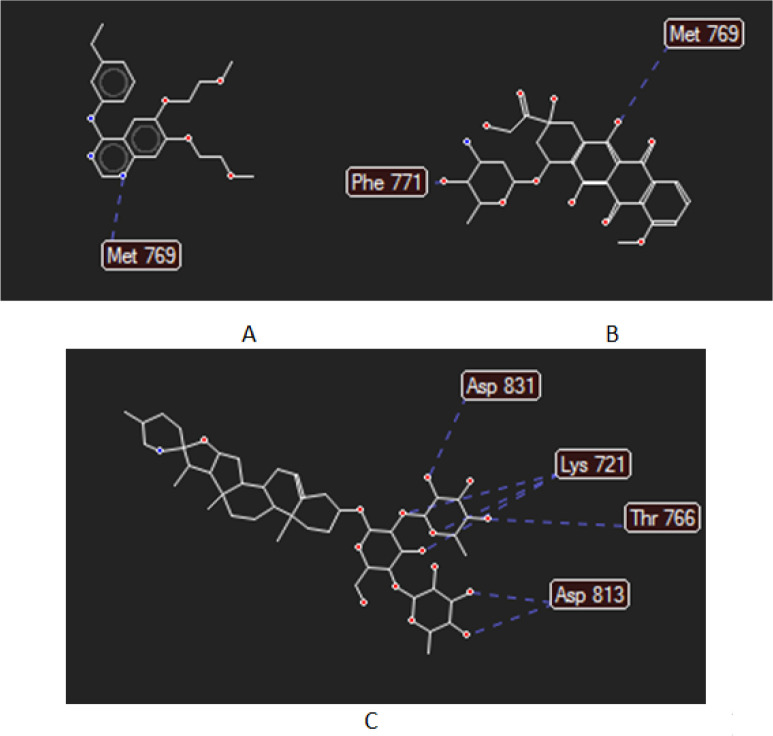
Interaction of Ligand (A) Erlotinib, (B) Doxorubicin and (C) Solamargine with Amino Acid Resides of EGFR

Molecular docking simulation has performed for the bioactive compound of *S. nigrum* L. such as diosgenin, sapogenin, solamargine, solanine and solanidine into E (PDB ID: 1M17). Each compound was positioned as ligands or inhibitors that inhibited the receptor performance. Coordinate for docking in position: x = 21.58; y = 0.40; z = 52.48 with radius 15 A. The result of docking score of each compound using Molegro Virtual Docker ver 6.0 program was represented at Table 2. The score was known or assumed as binding free energy between the target receptor, EGFR, with its ligands. Solamargine showed the lowest binding energy as -129 kcal/mol, lower than erlotinib, EGFR inhibitor agent, and doxorubicin, a chemotherapy drug. Negative values indicated the bond was more stable, which meant that the interaction between EGFR and the ligand might hinder these receptors’ performance in causing growth and survival cancer. Figure 5 displayed the interaction of ligand, erlotinib, doxorubicin, and solamargine with amino acid reside of EGFR. It was shown that erlotinib interacted with Met 769, doxorubicin with Met 769, and Phe 771, and solamargine with Asp 831, Thr 766, Lys 721, Asp 813 and Thr 830 of the EGFR ligand resides. Based on in vitro test results, it was known that the cytotoxic activity of *S. nigrum* L. extract was still lower than doxorubicin. It was likely due to the low solamargine levels in which *S. nigrum* L. crude extract contained complex secondary metabolic compounds.

**Table 1:** The IC_50_ Value of *S. nigrum* L. extract and Positive Control Doxorubicin against MCF-7 Cell

No	Extract/positive control	Cell lines	IC_50 _(μg/mL)
1	*Solanum nigrum* L.	MCF-7	40.77 (4.86)
2	*Solanum nigrum *L.	Vero	298.96 (27.28)
3	Doxorubicin	MCF-7	0.14 (0.05)

**Table 2:** Docking Score of Solanum’s Active Compounds with EGFR

Ligand	MolDock Score	Rerank Score	Hbond
Erlotinib	-123	-96	-2,6
Diosgenin	-103	-82	0
Doxorubicin	-93	-88	-3
Sapogenin	-110	-89	-1
Solamargine	-159	-129	-1
Solasodine	-102	-81	-2.5
Solasonin	-102	-82	-2.5
Daidzein	-82	-74	-5.1
Genestin	-108	-98	-16.2
Genestein	-86	-79	-7.2
Glycitin	-122	-111	-11.1

## Discussion

*Solanum nigrum* L. is known as Leunca in the local Indonesian name, which is used as vegetable or treatment the same illness locales. Chemical compounds contained in *S. nigrum* L. extract is responsible for its pharmacological activities. Previous research reported that *S. nigrum* L. extract contained alkaloid, flavonoid, saponin, tannin, protein, flavonoids, glycosides, saponin, sterol, terpenoid, and triterpenoid (Ashrafudoulla et al., 2016; Gogoi et al., 2012; Ramya et al., 2012). The content of total flavonoid compound in the different part of *S. nigrum* L. had been reported in the earlier study in which the ethanolic extracts of fruit, stem, and leaves of *S. nigrum* L. contained total flavonoid were 0.62, 1.32 and 2.55 mg QE/g dried extract, respectively (Alam et.al., 2012). Measurement of the phenolic compounds was carried out using Folin Ciocalteu reagent in which the hydroxyl group in the phenolic compound reacted with the Folin reagent to form a blue molybdenum tungsten complex after basification process with Na_2_CO_3_ solution, and the color can be detected using UV-Vis spectrophotometer at 725 nm (Chen et al., 2015). Phenolic compounds were claimed to demonstrate several pharmacological activities such as anti-inflammatory, antimicrobial, scavenging free radical compound (Chen et al., 2015), antigout (Ningsih et al., 2018), and anticancer through the caspase-mediated proapoptotic pathway and antiangiogenic effects (Carocho et al., 2013). It was reported that several isoflavone compounds of *S. nigrum* L such as β1-solasonine (fruit), diosgenin (fruit), inunigroside A (fruit), solamargin (whole), γ-solamargine (whole), khasianine (whole), solasodine (whole), solanigroside P (whole), solanigroside A (whole) (Kaunda et al., 2019). The level of isoflavon diosgenin in *Solanum nigrum* L. fruit was 4,000-12,000 ppm (Dweck AC, 2006). 

The selective cytotoxic properties of *S. nigrum* L. extract against other cancer and normal cells had been reported previously. The methanolic extract of *S. nigrum* L. fruit exhibited cytotoxicity activity significantly higher on HeLa cell than on Vero normal cell (Patel et al., 2009). The equivalent combination of *S. nigrum* L. ethanolic extract with doxorubicin performed synergism anticancer effect on T-47D breast cancer cells (Anindyajati et al., 2010). Both *S. nigrum* L. leaves and stems methanolic extracts also demonstrated as an anticancer agent on PC3 prostate and HeLa cervical cancer cells. They had a nontoxic effect on normal cell such as NIH T3T mouse embryo fibroblast and CC-1 rat hepatocyte cell (Moglad et al., 2014).

Study on cell cycle of previous corroborated studies such finding, particularly concerning cell cycle arrest activity of* S nigrum* L. toward other cancer cells. Forgoing research also reported the polyphenolic compound derived from the extract of ripe fruit *S. nigrum* L. arrested in various human prostate cancer cells (Nawab et al., 2012). Solamargine, a compound isolated from *S. nigrum* L., showed significant activities in inhibiting SMMC-7721 and HepG2 human hepatocarcinoma cell growth, inducing cell apoptosis, and also arresting cell cycle at the G2/M phase (Ding et al., 2012). Subsequently, a glycoalkaloid known as solasodine rhamnosyl glycosides, were secondary metabolites found in Solanaceae, specifically induces apoptosis in cancer cells (Cham, 2013).

In silico study is a computer simulation to predict such compounds by biological properties by binding activity toward the receptor. EGFR took the main part in all living cells, especially for differentiation, normal development, and proliferation (Xu et al., 2010). The inhibition of apoptosis in breast cancer is associated, such as EGFR and its signal transduction pathways (Xu et al., 2010; Garcia et al., 2006). The high EGPR expression in breast carcinoma confers an advantage of growth to the tumor cells. Due to its crucial role in cancer cell progression, the efforts to find new compounds with the mechanism of EGFR inhibition significantly increased. Besides the other genes, EGFR becomes one of the essential targets of novel antitumor agents. Thus, one of method in the breast cancer treatment has been evaluated by EGFR inhibitors in several studies (Masuda et al., 2012). The mechanism of standard chemotherapy drugs is addressed to inhibit the cancer cell proliferation, whereas disturbance of the essential pathways becomes the target of EGFR inhibitors.

These studies concluded that the ethanolic-70% extract of *S. nigrum* L. fruit demonstrated the ability to decrease cell viability, promote apoptosis and suppress cell cycle in breast cancer cell MCF-7, and showed nontoxic effect toward Vero normal cell. Molecular docking scores supported this activity. Some isoflavone compounds of *S. nigrum* L. were positioned as ligands or inhibitors that hindered the EGFR performance in which solamargine exhibited the lowest binding energy and lower than erlotinib. Phytochemical studies showed that this extract contained total phenolic and flavonoid compounds with a level of 1.545±0.080% and 0.212±0.002%. Glycitin was the highest level of isoflavone compound, namely, 375.0844 mg/100 g semisolid extract. In the subsequent studies, it was essential to measure the level of solamargine in this extract. Furthermore, this extract had the potential to be developed to become a selective natural anticancer agent.
